# A Concise Review of Recent Advancements in Carbon Nanotubes for Aerospace Applications

**DOI:** 10.3390/mi16010053

**Published:** 2024-12-31

**Authors:** Silvia Zecchi, Giovanni Cristoforo, Erik Piatti, Daniele Torsello, Gianluca Ghigo, Alberto Tagliaferro, Carlo Rosso, Mattia Bartoli

**Affiliations:** 1 Department of Applied Science and Technology, Politecnico di Torino, C.so Duca degli Abruzzi 24, 10129 Turin, Italy; giovanni.cristoforo@polito.it (G.C.); erik.piatti@polito.it (E.P.); daniele.torsello@polito.it (D.T.); gianluca.ghigo@polito.it (G.G.); 2 Consorzio Interuniversitario Nazionale per la Scienza e Tecnologia dei Materiali (INSTM), Via G. Giusti 9, 50121 Florence, Italy; mattia.bartoli@polito.it; 3 Istituto Nazionale di Fisica Nucleare, Sez. Torino, Via P. Giuria 1, 10125 Torino, Italy; 4 Faculty of Science, Ontario Tech University, 2000 Simcoe Street North, Oshawa, ON L1G 0C5, Canada; 5 Department of Mechanical and Aerospace Engineering, Politecnico di Torino, C.so Duca degli Abruzzi 24, 10129 Turin, Italy; carlo.rosso@polito.it; 6 Center for Sustainable Future Technologies—CSFT@POLITO, Via Livorno 60, 10144 Torino, Italy

**Keywords:** CNTs, CVD, nanomaterials, composites

## Abstract

Carbon nanotubes (CNTs) have attracted significant attention in the scientific community and in the industrial environment due to their unique structure and remarkable properties, including mechanical strength, thermal stability, electrical conductivity, and chemical inertness. Despite their potential, large-scale applications have been limited by challenges such as high production costs and catalyst contamination. In aerospace applications, CNTs have demonstrated considerable promise either in the form of thin layers or as reinforcements in polymer and metal matrices, where they enhance mechanical, thermal, and electromagnetic performance in lightweight composites. In this short review, we provide an overview of CNTs’ properties and structures, explore CNT growth methods, with a focus on chemical vapor deposition (CVD), and examine their integration into aerospace materials both as films and as multifunctional reinforcements.

## 1. Introduction

Among carbon-based compounds, carbon nanotubes (CNTs) represent a key class of materials. CNTs became famous after the Ijima article in 1991 [1], even if the first publications on these materials date back to the 1950s [2,3,4,5]. The unique structure of a single-walled CNT can be represented as a rolled-up single graphene layer, closed at one of its ends, and with a high aspect ratio (i.e., the ratio of its length to its diameter). This tubular structure is responsible for their specific mechanical, physical, and electrical properties in combination with their chemical inertness. In the last decades, CNTs have passed through different periods of alternating successes without ever finding a real large-scale application. The main challenges affecting the use of CNTs are their price and purity. CNTs are quite expensive, and their price is related to their purity. Few technologies [6,7] are able to produce this carbon material with high reproducibility on large-scale surfaces. Although this could be a limitation to their application to film materials, recently, other approaches for the production of CNT films have been proposed, such as the sandwich-structured thin film based on multiwall carbon nanotubes developed by Sun et al. [8]. Contamination of the CNT material, for example, due to the catalyst used to grow CNT films, can also be an issue when the application requires pure carbon material. As we discuss in this short review, catalysts are an essential component for CNT production, with the metals of group VIII of the periodic table (such as Fe, Ni, or Co) being the ones typically used for CNT growth. Such catalyst particles can be removed from the grown material via CNT purification, but such treatments can damage the CNTs’ structures, decreasing their properties (which will not be discussed here).

Beyond film applications, CNTs have gained considerable attention in the aerospace field due to their exceptional properties when embedded in composite materials. In polymer matrices, CNTs are employed as reinforcements to enhance mechanical strength, thermal stability, and electrical conductivity, enabling the development of lightweight yet high-performance composite materials [9]. Such CNT–polymer composites have found applications in structural components, electromagnetic shielding, and sensor technologies in aerospace [10]. Similarly, the integration of CNTs into metal matrices has opened new avenues for advanced aerospace materials. These composites exhibit improved mechanical strength, wear resistance, and thermal conductivity, making them ideal for demanding applications such as engine components and thermal management systems [11].

This short review is focused on providing the reader with an understanding of CNT production, properties, and applications. The review will begin with a description of CNTs’ general properties and structures to better focalize the topic for the inexperienced reader. The second part of the review is devoted to CNT growth, with a specific focus on the chemical vapor deposition (CVD) process because it is one of the most commonly used ways to produce CNTs. The final section of the review presents an in-depth discussion on the utilization of CNT-containing composites as reinforced multifunctional materials for aerospace applications.

## 2. Methods

This review is focused on CNT production and their use in composites for aerospace applications. It aims to show the key relevance of the issue, focusing on the properties of the CNT-based composites in relation to the CNT properties, including length, size, and procedures used for production. We searched for the most relevant research papers on this topic and limited our search to the following databases, which contain the most information in this field: Google Scholar, ScienceDirect, and SciFinder. Several keywords were used either alone or in combination, such as CNTs, CVD, CNT composites, aerospace, and CNT reinforcement. The last search was carried out on the 3rd of December 2024. By searching these databases, we found more than 1000 citations; 178 of them were selected based on two criteria: time of publication limited to the last 10 years and key information provided, including parametric studies of CNT properties.

## 3. CNTs’ Properties and Structures

**Figure 1:**
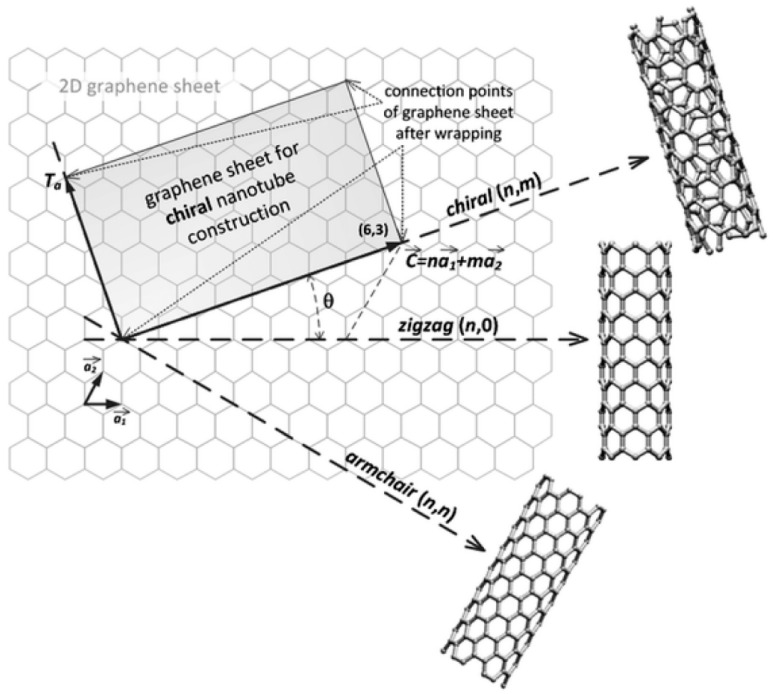
Folding of a graphene sheet according to the chiral vector $\vec{C}$, leading to the three main CNT structures known as armchair, zig-zag, and chiral. Reprinted from Sanginario et al. [13] (CC BY).

CNTs are an allotropic state of carbon that can be described as a cylindrical graphene sheet rolled up in a tubular structure according to the chiral vector $\vec{C}$ reported in Equation (1) [12].

(1) $$\vec{C}=n{\vec{a}}_{1}+m{\vec{a}}_{2}$$

where *n* and *m* are positive integers named chiral indices and ${\vec{a}}_{1},\;{\vec{a}}_{2}$ are primitive vectors of a graphene plane, as shown in Figure 1.

The chiral indices (n, m) can be used to define the three structures of a CNT according to the orientation of its longitudinal axis with respect to the hexagonal lattice. Three different structures (CNT types) can be identified, namely, zig-zag (m = 0), armchair (n = m), and chiral (n ≠ m ≠ 0). Chiral CNTs show the geometrical properties of enantiomeric molecules. Furthermore, Belin et al. [14] reported that the electronic properties of CNTs can be predicted based on their chiral indices and summarized in two different cases, as follows:

(i) |n − m| = 3q, q∈N, q ≠ 0
(ii) |n − m| = 3q ± 1, q∈N, q ≠ 0

In the first case, CNTs show a metallic behavior, while in the second one, they show semiconductor properties. Armchair CNTs always satisfy the first condition and thus show a metallic behavior.

**Figure 2:**
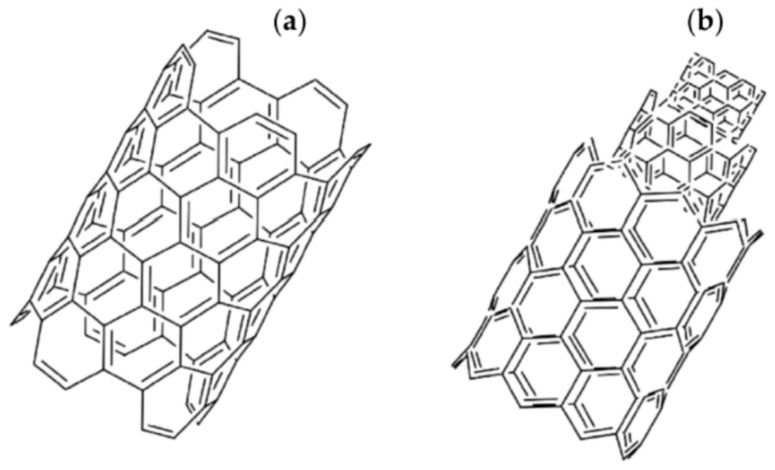
Longitudinal scheme of (**a**) SWCNT and (**b**) MWCNT. Reprinted from Giorcelli et al. [15] (CC BY).

CNTs can be composed of single or multiple graphene sheets, known as single-walled CNTs (SWCNTs) and multiwalled CNTs (MWCNTs), respectively, as shown in Figure 2.

SWCNTs have diameters ranging from 0.3 nm [16] to more than 1 nm [17], while MWCNTs can reach diameters of 100 nm [18,19]. CNT lengths are highly variable and depend on the production process; they can range from a few nanometers [20] up to half a meter [21]. Furthermore, CNTs can be either open-ended or closed, with half of a fullerene-type molecule [22].

Due to their unique sp^2^ carbonaceous hexagonal structure, CNTs display superior electrical, mechanical, and thermal properties, as reported in Table 1, which give them excellent material properties.

CNTs exhibit attractive electrical properties, such as ballistic transport. The electronic behavior of CNTs is related to their structure and morphology [30]. As discussed previously, the chirality of CNTs directly determines their electrical properties, leading to a metallic behavior for armchair configuration and to a semi-conductive behavior for zig-zag and chiral structures [31]. Therefore, the electrical behavior of SWCNTs is quite variable. The electrical behavior of MWCNTs is even more complex as they are formed by multiple concentric SWCNTs. Their structure is richer in defects and inter-layer interactions that generally lead to a higher resistance to electron flow [25,28,32,33]. However, they are more mechanically resistant than SWCNTs. Overall, SWCNTs demonstrate higher electron mobility and tunability of electrical properties, while MWCNTs have high mechanical robustness and the ability to transport high current densities (up to 10 A/cm^2^) [34]. Moreover, a key factor for CNTs’ electrical properties is the aspect ratio; for instance, in composite materials, CNTs with high aspect ratios make it possible to reach the percolation threshold at much lower CNT loading with respect to CNTs with low aspect ratios [34].

## 4. CNT Growth Through CVD

CVD is considered to be a low-cost process for the production of CNTs, and it is the most commonly used method for the deposition of CNT thin films. The most relevant features of this technique are the high yield of CNTs produced and the temperature requirements [22]. Furthermore, CVD enables great control over both the morphology and structure of the CNTs, leading to the growth of aligned CNTs [35,36] with reasonably low residence times [37].

### 4.1. CNT Growth Mechanism Through CVD

The mechanism of CNT growth during the CVD process is the key to the success of this approach for CNT production [35,38], as summarized in Table 2.

**Figure 3:**
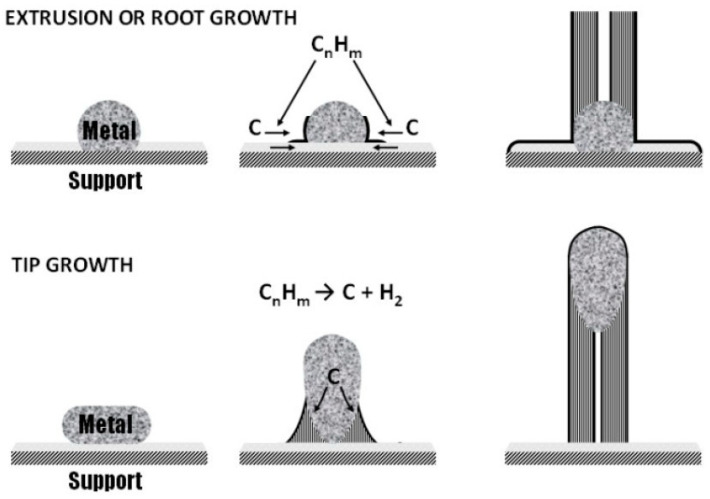
Mechanisms of CNT growth during CVD process: root and tip growth.

These procedures require the use of a metal catalyst, such as iron- [62], cobalt- [63], or nickel- [64] based ones, as they represent the best compromise between the process conditions, cost-effectiveness, and properties of CNTs produced [65]. As shown in Figure 3, CNT growth can proceed through two different mechanisms, namely, root and tip growth [66].

Both mechanisms involve the molecular decomposition of the carbon precursor, generally hydrocarbons, on the side of catalytic particle, leading to the formation of metal carbides that become supersaturated [67].

The root growth mechanism takes place when the adherence of the particle to the surface is strong so that the carbon precipitates from the top surface of the catalyst and the metal particle remains attached to the substrate during the growth of the filament [68].

Tip growth occurs when the adherence of the particle to the surface is weak so that carbon precipitation occurs at the bottom surface of the catalyst and the filament lifts the particle as it grows, capping the top of CNTs [69].

As discussed by Tessonnier et al. [70], the accepted model for CNT growth is based on vapor–solid–solid, in which it is possible to select the SWCNTs’ growth by reducing the size of catalyst particles as much as it is possible and simultaneously regulating the type and degradation rate of carbon precursors by tuning both the catalytic system and temperature [71,72,73].

### 4.2. State-of-the-Art CNT Growth Through CVD

**Figure 4:**
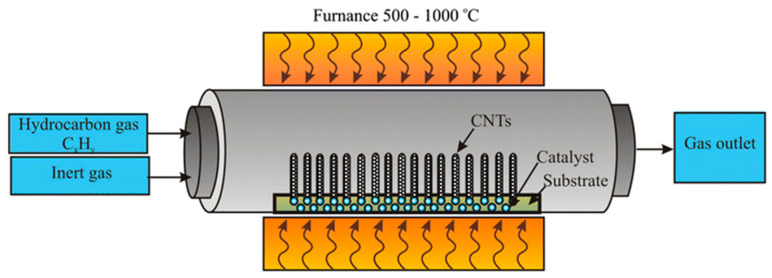
Simple scheme of CVD run in a tubular reactor, as reported by Zaytseva et al. [74] (under CC license).

CVD growth of CNTs can be performed by multiple approaches, but the most commonly used one is based on a tubular furnace containing the catalyst, which is supported onto a substrate and heated to between 500 °C and 1000 °C, as shown in Figure 4.

**Figure 5:**
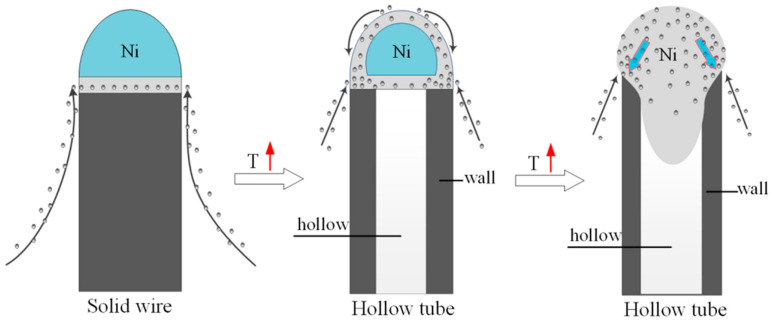
Conversion of ethylene into polycrystalline carbon nanowires and formation of CNTs according to the mechanism proposed by Pan et al. [75].

As reported by Pan et al. [75], temperature is a critical issue for the overall CVD process. The authors investigated the thermal degradation of ethylene onto nickel nanoparticles distributed in a porous Si_3_N_4_ ceramic material at 700 °C. By using this temperature, they were able to produce polycrystalline carbon nanowires with a solid core and basal planes of graphite nanosheets perpendicular to the wire axis. By increasing the temperature of the process to above 750 °C, the carbon structures evolved to CNTs with a tip growth mechanism, as shown in Figure 5.

The authors also reported that further annealing to 1000 °C converted the as-grown CNTs into highly crystalline ones.

#### 4.2.1. CNT Growth Mechanism Through CVD and SWCNTs

The challenge of the selective production of SWCNTs was also tackled by developing suitable synthesis methodologies, as reported by Ohashi et al. [39]. The authors developed a remote plasma CVD process for the production of SWCNT forests by using carbon monoxide as a carbon source, achieving the selective production of small-diameter SWCNTs.

Similarly, Anoshkin et al. [40] used a mixture of carbon dioxide and ethylene for the production of SWCNTs through aerosol CVD. The mixture used by the authors showed a different decomposition temperature and led to a final product with a reduction in sheet resistance from 7500 Ω/sq, when pure carbon monoxide was used as a precursor, to 291 Ω/sq, when deposition was performed at 1100 °C in the presence of ethylene.

Iakovlev et al. [41] reported an innovative spark-discharge aerosol CVD reactor using ferrocene as a catalyst precursor. The authors described the production pathways through two mechanisms: the first, named ex situ, proceeded while the spark-discharge generator formed the aerosol of nanoparticles, whereas the second, named in situ, occurred via the decomposition of the ferrocene vapor directly on the CNT growth zone, providing an in situ formation of the catalyst. The authors clearly showed that the ex situ activation provided an inferior activity of the catalyst nanoparticles due to their over-coagulation.

Cantoro et al. [76] conducted an interesting study on the production of SWCNTs at very low temperatures, ranging from 350 °C to 450 °C. The authors used ethylene as a carbon source, together with an iron/aluminum film supported onto silicon as a catalyst, producing a SWCNT carpet with a thickness of up to 500 μm.

Krasnikov et al. [42] developed a spark-discharge generator for scalable aerosol CVD synthesis of SWCNTs. The authors claimed that their method resulted in the separation of the processes of nanoparticle formation and CNT nucleation, leading to the independence between growth parameters and the diameter distribution of CNTs, therefore enhancing the scalability of the process.

Ikuno et al. [61] proposed an innovative approach based on low-pressure CVD for the production of aligned CNTs. The authors used pure ethylene and an iron nanoparticle catalyst at a residual pressure of 100 Pa, showing a preferential formation of vertically aligned CNTs. Lin et al. [77] extended this study by investigating both relatively high- (600 Pa) and low- (0.3 Pa) pressure CVD in a plasma environment. The authors showed an improved CNT growth rate of up to 1–3 μm/min at high pressure compared to a rate of 0.1 μm/min attained using low pressure. The authors ascribed the difference to an increment in depositing species under higher plasma pressure, suggesting that mass transport is the rate-controlling step of the CVD process.

Maghsoodi et al. [43] developed a novel continuous CVD process for SWCNT synthesis by using an iron floating catalyst and magnesium oxide particles by using methane. The advancement of this work was represented by the unique mechanism occurring in the hot zone of the reactor, where sublimed ferrocene vapors came into contact with magnesium oxide powder fluidized by methane, producing the catalyst in situ. An annular tube was used to enhance the ferrocene and MgO contact efficiency. Furthermore, the apparatus worked continuously, with CNTs collected at the bottom of the reactor. Nonetheless, the CNTs produced via this process were highly defective, as proven by Raman analysis, which showed an I_D_/I_G_ ratio of up to 10.

This drawback is overwhelmed by the industrial attractivity of the in-floating catalyst CVD processes even if OCSiAl obtained good results in the mass production of SWCNTs [78,79]. Accordingly, Ahmad et al. [44] conducted a systematic investigation on both monometallic (iron, cobalt, and nickel) and bimetallic (cobalt/nickel and cobalt/iron) catalysts used for this CVD procedure. The authors showed that iron-based materials were far more active than nickel- or cobalt-based ones. Interestingly, the authors reported that a bimetallic cobalt/nickel-based catalyst could promote the production of chiral SWCNTs in the chiral angle of 15° to 30° but did not dramatically shift the chirality of floating-catalyst CVD SWCNTs.

These results were in good agreement with those of Bahrami et al. [45], who studied the degradation of methane over floating iron oxide/magnesium oxide materials in the temperature range of 900 °C to 1000 °C.

Harutyunyan et al. [80] developed a mild-condition CVD process based on a metallic or oxide iron/molybdenum catalyst supported onto alumina and operating in the temperature range of 600 °C to 900 °C. The authors claimed a selective production of SWCNTs by using methane as a carbon source and a molybdenum load of up to 20 wt.% without any catalyst pre-activation by using hydrogen. They also reported that the optimum conditions were achieved by using an oxide form at 680 °C with a methane flow of up to 40 cm^3^/min. The SWCNTs produced had diameters ranging from 0.7 to 1.7 nm, without any evidence of MWCNTs formation. This approach could be tentatively scaled up to gram-per-hour production.

Cassel et al. [81] made additional progress for the large-scale production of CNTs through CVD. The authors reported the catalytic degradation of methane over an iron oxide/molybdenum oxide catalyst supported onto alumina to produce SWCNTs. The authors achieved a productivity of up to several grams per hour at 900 °C. The purity of the materials was far inferior to that obtained by Harutyunyan et al. [80], with bundles of SWCNTs partially covered by amorphous carbon.

Better results could be achieved by using alumina instead of silica, as reported by Su et al. [82]. The authors used also an iron/molybdenum catalyst supported onto a silica aerogel, which increased productivity, with the weight of the SWCNT produced being double that of the used catalyst.

Ahmad et al. [46] also found that sulfur used for the production of SWCNT-based transparent conductive films affected the yield but not the quality of CNTs produced.

Alternatively, several other combinations of metals can be used to exploit different growth rates, sizes, and shapes of CNTs produced [83,84].

Bouanis et al. [85] described CVD synthesis of SWCNTs by using ruthenium nanoparticles in a hot filament reactor. In this case, the authors established that properties such as the quality, density, and diameter of SWCNTs were related to the filament and growth temperature.

A more traditional approach was reported by Izadi et al. [47] using a fixed-bed reactor and a multimetallic catalyst (Co–Mo–MgO).

In addition to experimental setups, growth support deeply influences the CVD of CNTs, as shown by Kim et al. [48]. The authors described the CVD growth of SWCNTs over several metallic (aluminum/copper, copper, silver, thallium, and nickel/vanadium) substrates by using ethylene. The results showed a strong relationship between the metallic support and both the type and quality of the CNTs.

Pasha et al. [86] used a chromium/iron-based catalyst for the conversion of petroleum gas to CNTs in a hot filament reactor. The authors reported poor efficiency of the synthesis, with the formation of large amounts of amorphous carbon and a very low yield of highly defective CNTs. Nonetheless, a chromium film supported onto silicon was used by Liu et al. [87] for the production of CNTs under microwave plasma conditions. The authors further reported the possibility of growing CNTs directly on the reactor walls, paving the way for industrial-scale applications.

Rhenium catalyst was first used by Ritschel et al. [88] in a fixed-bed reactor for the production of a mixture of SWCNTs and MWCNTs. The high cost of the catalyst, together with the poor selectivity for one class of CNTs, slowed down the adoption of this approach, in favor of other techniques that are more reliable and that use cheaper materials.

Bhaviripudi et al. [89] first described the use of gold nanoparticles as a catalyst for the production of SWCNTs using ethylene at 800 °C. SWCNTs showed a very small diameter close to 2 nm and the quasi-absence of the D-band in Raman spectra. The D-band is a diagnostic signal in the Raman spectrum of CNTs [90] and is related to their diameter [91,92] and order [93]. The very low intensity of the D-band presented clear evidence of the high quality and small diameter of the SWCNTs produced using a gold catalyst. The authors also hypothesized that the active nanoparticle shell was mainly composed of gold oxide with traces of chlorine as residues of the precursor.

Wong et al. [94] used palladium thin films to produce CNTs after ammonia treatment, achieving an average diameter of up to 20 nm. Other noble metals such as ruthenium and platinum [95] could be used alone or in combination with iron [96] for the CVD process.

The other relevant parameter of CVD processes for CNT production is the carbon feedstock used for their synthesis. Almkhelfe et al. [49] used Fischer–Tropsch-derived gas for the production of a high-quality and well-aligned SWCNT carpet. The authors tested both cobalt and iron catalysts and found the optimum temperature conditions to be 850 °C and 750 °C, respectively.

Acrylonitrile combined with other hydrocarbon sources could also be used for the production of SWCNTs with uniform diameters and (n, m) chiral indices [50]. Using quantum chemical molecular dynamics simulations, the authors were able to describe the formation mechanism, which was based on acetonitrile-derived radicals that removed a hydrogen atom from the hydrocarbon molecule and formed an isocyanide species. By liberating hydrogen, the number of dangling bonds at the interface of the carbon-nucleating structure increased, leading to faster SWCNT nucleation kinetics.

Shandako et al. [97] also reported the use of ethanol as an efficient carbon precursor for the synthesis of SWCNTs at temperatures ranging from 750 °C to 1000 °C.

#### 4.2.2. CNT Growth Mechanism Through CVD and MWCNTs

Moving forward, double-walled CNTs represent an interesting bridge between SWCNTs and MWCNTs [98]. Kim et al. [99] reported the first comprehensive study on the topic, showcasing several features of these materials, ranging from thermal and electrical properties to inner-wall/outer-wall chemistry. Ci et al. [100] produced these materials through the CVD approach of double-walled CNTs by using a sulfur-doped floating iron catalyst at 1100 °C in an ethylene atmosphere, showing that both precursors and catalysts should be chosen wisely; they also showed that analog but not homolog systems lead to different outputs [75]. The authors reported the formation of CNTs with diameters ranging from 0.4 to 2 nm.

Compared to SWNT production, the synthesis of MWCNTs is far easier, and after thermal annealing at 2200–2800 °C, the produced materials are also highly crystalline [101]. Bansal et al. [51] compared the yield of MWCNTs between CVD and water-assisted production methods and reported a greater yield with the use of CVD processes.

The scalability of MWCNT production was investigated by Couteau [102] using an iron/cobalt catalyst supported onto calcium carbonate. The use of calcium carbonate involved a crucial advantage due to the absence of porosity, avoiding the formation of amorphous carbon during the nanotube growth. This led to a far easier removal from both the metallic particles and catalyst support through washes with diluted acid solutions without damaging the graphitic walls. Additionally, calcium carbonate decomposes at high temperatures with the release of carbon dioxide, decreasing the amount of material to remove after the CVD process.

Singh et al. [103] studied the scale-up of aligned MWCNTs on quartz reactors by using a ferrocene catalyst with relatively poor defect concentrations.

Continuous production was attained by Kunadian [104], but the produced materials showed structural defects, as described by other authors [105,106] who monitored the process through Raman spectroscopy.

Li et al. [107] ran a CVD directly on an aluminum foil in atmospheric plasma conditions at temperatures ranging from 600 °C to 900 °C. The authors showed the formation of tightly packed MWCNTs with a wall spacing of up to 0.34 nm and diameters ranging from 10 nm up to 47 nm.

Choe et al. [52] evaluated the influence of reagent stoichiometry on the yield and aspect ratio of MWCNTs obtained via acid-oxidized injection CVD, and they found the best set of conditions for keeping the CNT diameter constant during growth. The authors found a direct relation between iron injection concentrations and an increase in MWCNT diameter.

Das et al. [53] produced MWCNTs through microwave plasma CVD by using an iron catalyst, obtaining materials with an average diameter of up to 15–25 nm. Escobar et al. [54] instead used a simple thermal CVD and a catalytic process based on a thin-layer nickel catalyst, resulting in the production of CNTs rather than carbon fibers. Similar results were achieved by Fleaca et al. [55] by using a hot filament approach at 1 bar and 700 °C. The authors produced long fibrils with a diameter close to 1 μm, made by both MWCNTs and carbon fibers. Better results could be achieved by using a multimetallic catalyst with a high load of cobalt, as reported by Gromov et al. [56]. The authors produced MWCNTs with a diameter of 8 nm by using acetylene and a catalyst with 15 wt.% of cobalt and zirconium at only 650 °C.

Traditional ferrocene-based catalysts could be easily improved by adding nickel to improve the crystallinity of the CNTs due to the formation of regular catalyst particles of 10 nm that can boost regular growth. Some authors grew a thin film of CNTs on silicon and found that the optimal temperature was above 800 °C [108].

Das et al. [53] reported a high-yield MWCNT growth by using carbon dioxide/methane/hydrogen as feedstock under microwave plasma conditions at 300 °C with a CH_4_ + CO_2_ + H_2_ gas mixture. Catalysts based on iron nanoparticles have been grown via wet chemical processes at varying growth temperatures, and by controlling the catalyst iron nanoparticle synthesis, the authors achieved high control over the MWCNT diameter, reporting the selective production of MWCNTs with diameters ranging from 10 nm to 100 nm.

Nickel is an excellent choice for the production of MWCNTs through CVD, as reported by many authors [57,58], resulting in good yields at temperatures as low as 600 °C. Show et al. [109] used a triode-type plasma-enhanced CVD method for the production of aligned CNTs with a length of up to 3 μm at only 550 °C. Zhong et al. [60] used a combination of iron and titanium as a catalyst for the growth of MWCNTs onto titanium-coated silicon oxide, producing very thin layers of CNTs.

Another promising approach to the large-scale production of MWCNTs was reported by Lin et al. [58], who produced a smart precursor using thiophene coordinate with nickel. This material was treated at temperatures ranging from 600 °C to 900 °C, and good-quality MWCNTs were recovered from its decomposition. The authors claimed a yield of up to 93%, calculated as the ratio between the weight of CNTs and the weight of the catalyst.

Aerosol decomposition is another great tool for the CVD growth of MWCNTs. Since the pioneering research of Andrews et al. [110], thermal decomposition of aromatic hydrocarbon/metal-particle aerosol has played a central role in the advancement of the CVD technique [111,112,113,114].

Mayene et al. [115] produced an aligned MWCNT carpet with a thickness of up to 130 μm and with the average outer diameter of CNTs ranging from 10 to 200 nm through the pyrolysis of a benzene/ferrocene aerosol produced using a compressed gas-driven atomizer. The authors used temperatures ranging from 800 °C to 950 °C, achieving a good CNT yield at the lower temperature. Similarly, Kamalakaran et al. [116] produced thick and crystalline nanotube arrays using spray pyrolysis of an aerosol of ferrocene and benzene at 850 °C.

Meysami et al. [117,118,119] conducted comprehensive research on the use of different aerosol compositions, pyrolysis parameters, and catalyst effects on MWCNT production. The authors reported the synthesis of a large area (up to 90 cm^2^) of a millimeter-thick vertically aligned MWCNT carpet. Furthermore, they reached productivity of up to 14 g/h by scaling up a simple reactor.

Merchan-Merchan et al. [120] reported the formation of dense MWCNT aggregates produced without any catalyst addition by a continuous process carried out at atmospheric pressure in counterflow oxymethane diffusion flames. Similar procedures were reported by the authors for the production of aligned MWCNTs by applying an electric field of 1000 V/cm, with or without catalyst addition [121,122].

Aziz et al. [123] produced high-quality MWCNTs by an injection CVD method using ferrocene and toluene as carbon sources and investigated the effect of temperature on structural defects, impurities, thermal stability, sample morphology, and average diameter. The authors reported an appreciable change in quality, average diameter, and purity with an increase in temperature from 700 °C to 850 °C. At 700 °C, the authors observed MWCNTs with an I_D_/I_G_ ratio close to 0.2, with outer and inner diameters of 25 ± 6 nm and 7.8 ± 1.5 nm, respectively.

Recently, Zhang et al. [124] grew MWCNTs on pine nut shell biochar at 600 °C. Even if the CVD process produced a poor-quality material, where MWCNTs were mixed with carbon black, the study is the first one to examine sustainable CNT production.

Ozkan et al. [125] grew MWCNTs on hydrophobic sand particles at a high temperature with iron-based catalysts. MWCNTs were produced with good yields, but they required strong acidic conditions for purification, resulting in the insertion of several functional groups.

Alternatively, MWCNTs could be produced from waste plastic by using several catalysts (i.e., nickel/molybdenum onto manganese oxide, nickel [126] or iron/manganese [127], nickel onto alumina [128], and nickel/molybdenum onto magnesium oxide [129]) and temperatures close to 800 °C.

Furthermore, Xu et al. [130] developed a CVD process without any catalyst by using-(2-(trans-3,4-Cyclohexanediol)ethyl)-Heptaisobutyl as a carbon source, producing CNTs with an average diameter of up to 16 nm.

Liu et al. [131] achieved promising results by using a lanthanum oxide catalyst to produce helix-shaped MWCNTs.

## 5. CNTs for Aerospace Applications

### 5.1. CNTs in Thin Films

The aerospace industry requires novel and innovative materials that are lightweight and have outstanding mechanical, thermal, and electrical properties. Thanks to their unique properties, nanostructured CNT thin films represent a potential opportunity for advanced applications in the aerospace sector. CNT films are guaranteed to have superior performance as well as a lower mass, a critical aspect in aerospace applications. In this review paper, we focus on studies that have been conducted in the last few years and that have examined CNT thin films for advanced applications in the aerospace sector, such as EMI shielding, lightning strike protection, structural health monitoring, and mechanical toughening of composite structures.

#### 5.1.1. Lightning Strike Protection

During its operational life, an aircraft is exposed to severe conditions, such as flying in the presence of lightning activity. Consequently, the aircraft’s structure must be protected against strong electrical and thermal shock. Lightning strike protection strategies typically involve the use of metallic meshes that are able to safely dissipate lightning energy. In recent years, CNT films have emerged as an alternative to traditional materials. Xia et al. [132] reported a method for the preparation of a CNT film modified with silver particles by electrophoretic deposition. The method allowed researchers to create films with a thickness of approximately 50 µm, depending on the parameters of the electrophoretic deposition process. Silver-modified CNT films were integrated into carbon fiber-reinforced polymer (CFRP) laminates. Simulated lightning strike tests were conducted to evaluate the performance of these films in comparison to conventional materials (copper mesh laminates). CNT films exhibited good mechanical performance after the strike, better than the copper mesh laminates. Moreover, the integration of CNT films into laminates without adhesives led to minimal delamination, even after high electrical and thermal stresses.

In a recent work by Zhu et al. [133], the researchers investigated the use of superaligned CNT films for the protection of aeronautical structures. Superaligned CNT arrays were prepared using the CVD method. After a multi-step method, CNT films were stacked to form a 30 µm thick superaligned film with high density and electrical and thermal conductivity. The structure was embedded as the external layer in a composite laminate made of a dielectric core (zirconia fibers) and a structural CFRP layer. Simulated lightning tests were carried out in order to evaluate the protection capability of the CNT film. After the test, which was carried out using the lightning strike approach by applying up to 100 kA, the structure exhibited no internal damage, demonstrating the effectiveness of the CNT film, which was capable of high energy dissipation and structural protection.

A similar approach was used by Bai et al. [134]. In their work, superaligned CNT films were produced, densified on quartz fiber prepregs, and embedded on a CFRP layer. A 100 kA lightning strike test was carried out. As demonstrated by CNT films on zirconia fibers, the combination of CNT films and quart fiber prepregs is an effective solution for structural protection. The results showed that the integrity of the CFRP layer was preserved, with only external damage having occurred, highlighting the potential of these structures to replace the current materials.

Another work proposed a CVD process for the deposition of CNT films on carbon fibers [135]. The authors suggested using proper process parameters to avoid the degradation of CFRPs’ mechanical properties. They achieved substantial improvement in the electrical properties of layered materials, which increased the surface electrical conductivity by 300%, thus enhancing lightning strike protection.

#### 5.1.2. Sensing Applications

CNT films’ sensing capabilities have been investigated in various fields; this technology exhibits efficient detection for temperature, gas, etc. [136,137,138,139,140]. In the aerospace sector, CNT thin films can play a leading role in in-life sensing. A recent work by Karlsson et al. [141] explored the use of vertically aligned CNTs as sensors. In this study, vertically aligned CNT forests were synthesized by CVD on stainless steel substrates and transferred on Glass Fiber Reinforced Polymer (GFRP) prepregs. Subsequently, the CNT film, with a thickness of about 20 µm, was integrated into the structure during the vacuum-assisted infiltration process and subjected to curing. In situ monitoring of the electrical parameters of the film allowed the detection of transitions typical of curing processes, which were confirmed by other analyses. At the end of the thermal process, the sensor remained integrated into the structure, thus ensuring its function during the operational life of the component.

Another work focused on the integration of a similar structure into structural composites for strain and temperature sensing [142]. In this work, researchers grew vertically aligned CNT forests and deposited them onto GFRP prepregs with the method presented in [127]. The authors prepared two different configurations, varying the amount of embedded CNT forests (single-layer or double-layer), with the CNT forest thickness varying from about 20 µm to 50 µm. The samples were tested for electrical, strain, and temperature sensing to evaluate their capabilities. The CNT films exhibited a negative resistance coefficient and a linear piezoresistive behavior; these results show that CNT forests are a suitable solution for both temperature sensing across a wide range of temperatures (from −70 °C to 180 °C) and in situ monitoring of aerospace components during their lifecycle.

A novel hybrid sensor made of MXene and a CNT film was developed to monitor the health of fiber-reinforced composites [143]. MXene powders and MWCNTs were dispersed in two different solutions. Then, single layers of MXene sheets and CNTs were alternatively deposited and dried to form a hierarchical structure. The final film thickness was approximately 20 µm. The film was embedded in a composite structure during the resin process, and by evaluating its electrical parameters, it was found that the film demonstrated the capability for real-time monitoring of the curing process. Then, it underwent mechanical tests to evaluate its ability to monitor structural health. After tensile and bending tests, the film exhibited a Gauge factor of 115.3 and long-term durability, highlighted by stable resistance behavior after 1000 tensile cycles.

#### 5.1.3. EMI Shielding Applications

CNT films exhibit excellent electrical conduction, making them suitable for shielding applications in the microwave frequency range. Zhang et al. [144] focused on the development of lightweight and efficient solutions for EMI shielding applications. In their study, they proposed a multi-step process to create a thin and robust film based on carbonaceous materials. They synthesized CNT films via CVD, which were subsequently purified and densified by compression. A nanometric coating of glassy carbon was then deposited on CNT films via CVD. The performance of the samples was verified after exposure to harsh conditions (such as corrosive agents and high temperatures). The materials were tested to evaluate several properties, such as mechanical properties and EMI shielding behavior. Excellent results for EMI shielding applications were exhibited by a 2 µm thick treated film (EMI shielding effectiveness greater than 60 dB from 8 GHZ to 12 GHz). Good results were also exhibited by samples exposed to high temperatures: a 7 µm thick film showed a minimal decrease in EMI shielding efficiency (SE) (less than 5 dB) after 7 days at 200 °C. Moreover, these materials also exhibited Joule heating capabilities, demonstrating their potential for anti-icing applications.

Wan et al. also reported interesting results for CNT films in EMI shielding applications [145]. They presented a synthesis method based on CVD with a floating catalyst to produce pristine CNT sheets. Then, these sheets were purified and densified via acid treatment. The process led to strong interactions between MWCNTs, which resulted in a compact and conductive network of CNTs. The researchers produced films with a thickness ranging from 1.85 µm to 14.67 µm. EMI SE ranged from 51 dB for 1.82 µm thick films to an impressive 101 dB for 14.67 µm thick films over a wide range of frequencies. Moreover, mechanical tests showed excellent properties for densified CNT films, whose maximum tensile strength was reported to be 822 MPa.

Chen et al. presented an efficient method to grow aligned CNT films by a standard CVD process, followed by the integration of the carbon fiber laminate through a vacuum bagging process [146]. The study reported high surface conductivities, making those materials suitable for anti-static applications, EMI shielding, and lightning strike protection. The researchers highlighted that the materials have great potential for moving to production lines for industrial development since the technology is based on simple and currently used techniques, such as vacuum bagging.

Hybrid films based on stacked MXene and CNTs were prepared by an alternating vacuum filtration method [147]. In this work, the authors reported a maximum shielding effectiveness of 61.3 dB for a 12 µm thick sample.

Thin and flexible hybrid films based on CNTs and silicon carbide were developed by Sun et al. [148]. CNT hybrid sheets were stacked to form films with a thickness ranging from 30 µm to 70 µm and were then tested. The authors reported an EMI shielding effectiveness of 73 dB over a wide range of frequencies. Moreover, the film showed ablative resistance after exposure to high temperatures (up to 973 °C).

A study on hybrid films based on MXene and CNTs was also carried out by Hassan et al. [149]. They performed a modified CVD process to synthesize CNT films. Exfoliated MXene was deposited on a CNT film via vacuum-assisted filtration, a process that promoted strong interfacial bonding. The best EMI shielding performance of 72 dB was achieved by a 15 µm thick film in the 8.2–12.4 GHz frequency range. Further analysis demonstrated thermal camouflage ability at infrared frequencies.

Kim et al. grew CNTs directly on carbon fibers and glass fibers by a multi-step process involving CVD [150]. The researchers varied growth conditions to obtain different film thicknesses. After an integration step into a matrix, electromagnetic performances were evaluated. CNT-coated CF reported an EMI SE exceeding 70 dB. The authors stated that the developed process offers a scalable solution for the efficient production of shielding materials.

#### 5.1.4. De-Icing Applications

Hong et al. [151] explored the feasibility of the use of electrothermal films for random de-icing applications while maintaining transparency to radio frequency signals, a crucial aspect for ensuring good air–ground communication. In their study, MWCNTs were treated in solutions and spin-coated to produce thin films. Electromagnetic tests showed radio frequency transmittance that was greater than 80% in the 8.2–12.4 GHz frequency range. Moreover, thermal tests revealed a saturation temperature of 160 °C at 80 V, demonstrating good heating capability. De-icing tests validated CNT films’ potential for de-icing applications at −20 °C in less than 60 s.

#### 5.1.5. Mechanical Toughening of Composite Structure

A critical challenge related to composite materials in aeronautical structures is delamination. It consists of a failure mode where different layers of a composite structure separate under stress. External factors and work cycles can accelerate the process, limiting the operational life of the component. Several strategies have been explored to overcome this problem. Recently, CNTs exhibited great potential as a solution for next-generation aeronautical structures due to their mechanical properties and their ability to improve interfacial bonding while maintaining lightweight structures.

In a recent paper, Cao et al. prepared high-purity CNT forests (>99%) with a low thickness (≈100 nm) via CVD [152]. MWCNT forests were densified and integrated into a composite structure made of a PVC core and GFRP fabric, which were applied as a film on both sides. CNT films were placed as a reinforcing layer between PVC and GFRP. Mechanical tests were carried out to evaluate different fracture modes and interfacial strength. They reported strong improvements with two CNT reinforcing films at the PVC/GFRP interface. These samples achieved an increase larger than 125% for both mode I and mode II fracture toughness and an enhancement of over one and a half times for interlaminar shear strength. Moreover, numerical analysis reported a transition from brittle to ductile fracture mode.

A study on thin CNT veils as a toughening agent for CFRP layers was conducted by Ou et al. [153]. The authors used a floating-catalyst CVD process to deposit a thin, highly porous, and interconnected network of CNTs characterized by a thickness of approximately 30 µm. The thin film was incorporated into a multilayered structure. Mechanical analysis demonstrated an increase of 60% in mode I fracture toughness compared to the unreinforced laminate. SEM analysis highlighted the presence of a multimode crack propagation path promoted by the CNT veil, which effectively toughened the structure.

Two morphologies of vertically aligned CNTs, nanostitch and buckled nanostitches, were explored as interlaminar layers between polyimide prepregs [154]. CNTs prepared via thermal catalytic CVD were incorporated between PI sheets to characterize their mechanical behavior. A nanostitch-CNT film with a thickness of 10 µm exhibited the best performance, with improvements of 30% for both initiation toughness and steady-state toughness. The results were confirmed by SEM analysis, which showed crack bifurcation in the intralaminar region. This behavior effectively redistributed stress inside the reinforcing layer.

A study by Li et al. focused on the effect of CNT films on CFRP laminates for flexural properties [155]. A floating-catalyst CVD process was carried out to form 30 µm thick layers of CNTs. The films were interposed between CFRP laminates at specific positions and cured together. Different configurations were investigated, and experimental analysis showed that four-layer-CNT films resulted in increases of around 14% for both flexural strength and modulus. CNT films provided an effective way to ensure bridging between adjacent layers and deflect crack propagation, improving the interfacial performance of CFRP laminates.

A similar work was conducted by Fu et al. [156], who investigated the effect of vertically aligned CNT forests on unidirectional CFRP laminates. CNT films with a thickness ranging from 5 µm to 15 µm were interleaved into laminates and cured. Fracture tests highlighted enhancements of 61% with the 5 µm CNT film (mode I) and 67% with the 15 µm CNT film (mode II).

Vertically aligned CNTs were grown by Yang et al. via a CVD process directly on a carbon fiber surface [157]. In order to guarantee better interfacial adhesion between CNT-modified CF layers, a pre-coating treatment with acetone-diluted epoxy resin was performed. Composite laminates were prepared via compression molding and then tested. Flexural analysis showed an increase of 27% for flexural strength, with a transition in failure mode from delamination to crack propagation over the thickness of the sample. The result indicates effective reinforcement due to the CNT film at the interface.

A comparable pre-coating process was conducted by Cheng et al. [158] for the enhancement of adhesive bonding between a titanium alloy and CFRPs, in which a CNT film was interposed. In their study, a CVD process was conducted to grow a dense network of vertically aligned CNTs on an anodized titanium substrate. The substrate was joined to a CFRP composite. Mechanical tests revealed a 123% increase in shear strength and a transition from adhesive (in untreated composites) to cohesive failure (in CNT-based composites). Zhu et al. proposed a method to prepare superaligned CNT films via CVD on a silicon wafer [159]. The authors performed an acid treatment to enhance CNT film properties, leading to the collapse of the 3D structure into a 2D network. The film was incorporated into a polyamide amine for mechanical tests. The incorporation of acid-treated films in polyamide led to a huge improvement of 420% in toughness in comparison to superaligned CNTs. Impact tests demonstrated optimal energy absorption capabilities for the film, with performances comparable to conventional materials like steel and Kevlar. The researchers highlighted the multifunctionality of the film, such as EMI shielding, anti-icing and de-icing properties, and UV protection.

### 5.2. CNT-Based Composites

The development of advanced aerospace materials has resulted in significant progress but also faces substantial challenges. Carbon nanotubes, with their remarkable combination of mechanical, electrical, and thermal properties, have also emerged as a leading choice for reinforcing epoxy-based composites, revolutionizing the design and functionality of aerospace structures [10]. Additionally, CNTs have found applications in metal matrices, where they enhance properties such as strength, stiffness, and thermal conductivity, making them versatile for various aerospace components [160].

The bulk dispersion of CNTs requires a different production approach, in which CNT arrays are not as suitable as the unsupported ones. Accordingly, much of the industrial research has been focused on continuous production methods that do not require supports and are able to operate under fluidizing bed conditions [161,162,163] or using batch procedures such as arc discharge [164] and plasma-assisted techniques [165].

#### 5.2.1. Polymer Matrix Composites

In aerospace, the electrical and thermal performance of materials are critical. Epoxy/CNT nanocomposites are particularly promising in this regard. CNTs exhibit exceptional electrical conductivity, allowing their incorporation into epoxy matrices to enhance conductivity significantly. This property is essential for EMI shielding and electrostatic charge dissipation, which are both vital for the safety of electronic systems in modern aircraft [166]. The excellent EMI shielding properties of CNT/epoxy nanocomposites are due to their outstanding electrical conductivity and lightweight nature. Traditional materials like aluminum are effective but increase the overall weight of aerospace structures significantly. Studies have demonstrated that a CNT loading of just 3–5 wt.% in an epoxy matrix can achieve EMI shielding effectiveness exceeding 40 dB, outperforming conventional materials while reducing the structural mass [167]. Furthermore, the electrical properties of these composites can be tuned by modifying the CNT alignment or surface functionalization, enabling tailored performance for specific aerospace applications [168]. For example, functionalized CNTs integrated into epoxy matrices provide better electron transport pathways, which is crucial for maintaining the reliability of avionics in high-interference environments [160].

Thermal control remains a critical challenge in aerospace engineering, particularly for components exposed to extreme temperatures such as cryogenic tanks and hypersonic vehicles [160]. The intrinsic thermal conductivity of CNTs, which is among the highest, provides effective heat dissipation in epoxy composites. This is especially valuable in high-performance aerospace components exposed to extreme temperature variations, such as those encountered in space or during reentry. For example, composites with CNT–cellulose fillers exhibit an electrical conductivity of up to 100 S/cm, tensile strengths of 156–278 MPa, and strain capabilities of 3.7–7.0% [169]. CNT/epoxy nanocomposites exhibit a significant increase in thermal conductivity, up to 200% higher than neat epoxy, when the CNT content reaches 1–5 wt.% [10]. This improvement helps to mitigate thermal stress and enhances the durability of structures subjected to repeated thermal cycling, such as those found in reusable launch systems. Functionalized CNTs further enhance thermal performance by improving heat dispersion within the matrix, ensuring uniform heat dissipation and minimizing the risk of localized thermal damage [170].

A significant challenge in the development of hypersonic aircraft is managing the extreme thermal stresses encountered during atmospheric reentry, where temperatures can exceed 1500 °C. Gohardani et al. [171] reported that CNT/epoxy nanocomposites offer a solution to this problem by providing superior thermal conductivity and mechanical stability. The integration of CNTs within the epoxy matrix reduces thermal gradients across the structural components, thereby minimizing thermal stress concentrations that could lead to material failure. Furthermore, the CNT-enhanced epoxy shows improved resistance to ablation, which is a critical feature for protecting the outer surfaces of hypersonic vehicles during prolonged exposure to high-velocity airflow and intense heat. Their research highlighted how functionalized CNTs with optimized aspect ratios contributed to effective heat dissipation, ensuring a more uniform temperature distribution across the composite. This enhancement significantly delays the degradation of materials, extending the operational life of critical components, such as leading edges and control surfaces. The study also showed that precise control over CNT dispersion and alignment was key to achieving these improvements as poor dispersion could lead to localized weaknesses or reduced thermal performance.

#### 5.2.2. Challenges for Large-Scale Adoption

While CNT/epoxy composites offer tremendous potential, several challenges remain. Bulk production of CNTs with consistent quality, achieving optimal dispersion, aligning CNTs within resistant polymers, and ensuring robust adhesion at interfaces are ongoing technical hurdles [172]. Moreover, concerns about the environmental and health impacts of CNTs, particularly their toxicity, require careful consideration [173].

Weight reduction remains a primary objective in aerospace engineering due to its direct impact on fuel efficiency, payload capacity, and overall costs. CNT/epoxy composites contribute significantly to weight savings without compromising structural integrity. These composites exhibit a high strength-to-weight ratio, making them indispensable for applications in both commercial and military aviation [174,175].

Aircraft like Boeing’s 787 Dreamliner and Airbus A350-XWB exemplify the adoption of CNT-enhanced composites. Boeing’s 787 uses over 50% carbon-based materials, primarily in its wings and fuselage, while the Airbus A350-XWB incorporates 39% composites [176,177]. Similarly, military aircraft, such as the V-22 Osprey Tilt-Rotor and Tomahawk missiles, comprise CNT/epoxy composites due to their lightweight and high-strength characteristics. In some designs, up to 70% of an aircraft’s total weight is attributed to CNT/epoxy composites, underscoring their critical role in modern aviation [177].

One of the most significant challenges in utilizing CNTs in aerospace-grade composites is achieving uniform dispersion within the epoxy matrix. CNTs naturally tend to agglomerate due to strong van der Waals forces, leading to inconsistent material properties [172]. In order to address this, researchers have developed techniques such as chemical functionalization, which introduces reactive groups onto the CNT surface, enhancing compatibility with the matrix and promoting better load transfer [178].

For instance, functionalized CNTs wrapped in intumescent flame retardants have demonstrated improved dispersion and flame retardancy in polypropylene composites [178]. Similarly, in situ polymerization methods have shown success in controlling the diameter of functionalized CNTs, achieving dimensions between 20 and 90 nm for enhanced material integration [178].

Fire safety is a critical consideration in aerospace applications, where material performance under high temperatures can dictate the success of a mission. Studies on CNT/epoxy composites reveal that while these materials generally offer improved thermal stability and flame retardancy, their effectiveness depends on factors such as CNT concentration, aspect ratio, and dispersion quality [172]. For example, incomplete dispersion can lead to inconsistencies in flammability behavior, highlighting the need for further research [172].

The flame-retardant potential of CNT/epoxy composites is particularly promising when combined with other nanomaterials, such as aramid fibers or glass. These additives not only enhance their thermal stability but also contribute to the mechanical robustness of the composites [177].

The adoption of CNT/epoxy nanocomposites is already evident in numerous aerospace applications. Lockheed’s F-35 uses these materials in wingtip fairings, and NASA employs them in space shuttles for their superior thermal and mechanical properties [173]. Beyond structural components, CNTs are integrated into solid-fuel missiles and satellite rocket motor casings to withstand extreme operational environments [177].

NASA’s roadmap for CNT utilization underscores their transformative potential in aerospace. The envisioned benefits include reduced vehicle mass, improved damage tolerance, greater durability, and enhanced thermal protection [166]. Additionally, CNTs are being explored for self-healing materials and energy production systems, promising innovative solutions for future aerospace challenges [166,173].

#### 5.2.3. Metal Matrix Composites

Metal matrix composites are also extensively adopted in space and in advanced electronics because of their excellent properties, such as remarkable strength, hardness, and high elastic modulus [160,179]. The incorporation of CNTs into metal matrix composites (MMCs) holds immense promise due to the extraordinary mechanical, thermal, and electrical properties of CNTs. These properties, such as high strength, thermal conductivity, and electrical conductivity, make CNTs ideal candidates for reinforcing metal matrices. However, some challenges persist, particularly achieving the uniform dispersion of CNTs within the matrix and maintaining strong interfacial bonding between the CNTs and the metal. The tendency of CNTs to agglomerate during processing necessitates the use of specialized fabrication techniques. Merino et al. [180] focused on employing high-shear mixing and sonication to achieve better dispersion of CNTs in aluminum-based composites. They found that functionalizing the CNT surfaces significantly improved the interaction between the CNTs and the matrix, leading to more effective load transfer and enhanced mechanical properties. Similarly, Trinh et al. [181] explored ball milling as a technique to break up CNT clusters in aluminum, improving the tensile strength and hardness of the composite materials.

In addition to these approaches, Ma et al. [169] have investigated the use of additive manufacturing to fabricate CNT-reinforced MMCs. Their work showed that selective laser melting (SLM) allowed for precise control over the dispersion and orientation of CNTs within the matrix, leading to composites with superior mechanical properties and reduced porosity. This suggests that additive manufacturing holds significant potential for fabricating complex geometries in CNT-reinforced MMCs, although challenges remain in controlling CNT alignment and distribution.

Another notable issue is the balance between mechanical strength and ductility in metal matrix composites [182]. Despite their enhanced rigidity and strength compared to unreinforced metals, MMCs often exhibit reduced ductility. For aerospace applications, a minimum ductility of 3–5% is required, which remains a key challenge, alongside difficulties in machining, joining, and bonding MMC components [183]. The incorporation of CNTs as reinforcements in MMCs has shown promise in mitigating this issue. CNTs not only enhance the mechanical strength of the composite through load transfer mechanisms but also contribute to improving ductility due to their unique combination of strength and flexibility. Studies suggest that their nanoscale dimensions and excellent interfacial bonding can help distribute stress more evenly, thereby reducing the brittleness often seen in traditional MMCs.

Structural repair is another critical challenge, particularly for composite materials used in aerospace structures. While bonding technologies for composite repair can transmit sufficient stress and enhance joint efficiency, existing techniques still face limitations, especially under variable mechanical loading conditions. CNT-based adhesives and coatings have demonstrated the potential to improve repair durability and load transfer by enhancing the mechanical properties of the bonding interface. Their high surface area and exceptional thermal and electrical conductivity can also provide additional benefits in the context of structural repairs [184].

Space vehicle engines, designed for extreme temperatures, require materials that maintain microstructural stability, resist high-temperature creep, and endure fatigue under variable environmental conditions. The design of these components involves addressing corrosion, oxidation, and crack propagation. CNTs can provide effective reinforcements to improve the high-temperature stability and oxidation resistance of MMCs. Their ability to reduce crack initiation and propagation through crack-bridging mechanisms has been highlighted in recent studies, which also point to their role in enhancing creep resistance by impeding dislocation motion [185].

Surface treatment and coatings present additional challenges as composite usage expands in aerospace. Space shuttles and aircraft are subject to dynamic environmental conditions, including corrosion and oxidation. Coatings must enhance wear resistance while maintaining adhesion and thermal conductivity. However, currently used materials like aluminum, titanium, and their alloys have poor corrosion and wear resistance, particularly in high-altitude applications [186].

Fatigue cracking remains the predominant cause of structural failure in aircraft, necessitating meticulous material selection and design to mitigate this issue. Factors such as material microstructure, environmental exposure, and surface finish significantly influence crack initiation and propagation. The addition of CNTs to MMCs has been shown to reduce fatigue crack growth rates through mechanisms such as crack deflection and energy absorption. These properties, coupled with the ability of CNTs to enhance toughness, make them ideal candidates for improving the fatigue resistance of aerospace materials [187].

For defense applications, weight reduction remains a major focus in material innovation. Advanced composites and surface treatments play a pivotal role in the design of lightweight yet durable military aircraft and armored vehicles. Despite recent advancements, there remains a pressing need for optimized manufacturing methods and technologies to meet the growing demands of defense applications [182].

Sintering is one of the most common methods used for the production of CNT-reinforced MMCs. In this technique, CNTs are mixed with a metal powder and then heated to a temperature below the melting point of the metal, causing the particles to bond together [188]. While sintering is a relatively straightforward and cost-effective process, it often faces challenges in terms of achieving uniform dispersion of CNTs [189]. This is because CNTs tend to agglomerate due to van der Waals forces, making it difficult to uniformly distribute them throughout the matrix. Hot pressing and hot isostatic pressing (HIP) are advanced sintering techniques that apply pressure during the sintering process, promoting better particle packing and reduced CNT aggregation [180,190]. These techniques also improve the interfacial bonding between CNTs and the matrix, which is critical for improving the mechanical properties of the composite.

Casting is another widely used method for the production of CNT-reinforced MMCs. This process involves melting the metal matrix and then adding CNTs to the molten metal [191]. The composite is then allowed to cool and solidify into a desired shape. One of the significant challenges in casting is the poor dispersion of CNTs, which tend to form agglomerates in the molten metal. To address this issue, researchers have employed techniques like stir casting, where the molten metal is stirred during the process, and ultrasonic casting, where ultrasonic waves are applied to break up CNT agglomerates and improve their dispersion in the matrix [192]. These methods have been found to significantly improve the mechanical properties of CNT-reinforced MMCs, particularly in terms of tensile strength, hardness, and wear resistance [193].

Chemical vapor deposition is another technique used to create CNT-reinforced MMCs. CVD involves the growth of CNTs directly on the metal particles or within a metal matrix using carbon-based gases and a catalyst [194]. This method allows for better control over the structure and quality of the CNTs, ensuring that they are uniformly distributed and well-bonded to the metal matrix. CVD-grown CNTs provide superior interfacial bonding between the CNTs and the matrix due to the chemical bonding that occurs during the growth process. This improves the mechanical and thermal properties of the composite.

The impregnation technique involves the infiltration of CNTs into a molten metal or polymer matrix [195]. This method allows for better control over the CNT distribution and the interfacial bonding between the CNTs and the matrix. One variation of this method is resin-based impregnation, where CNTs are dispersed in a resin and then infiltrated into the metal matrix. This technique has been used to fabricate composites with improved mechanical properties, including increased tensile strength and fatigue resistance. Molding is another technique that involves pressing a mixture of CNTs and metal powders into molds, followed by heating to form a solid composite [196]. This method is ideal for creating complex-shaped parts that require high performance and durability.

In addition to traditional methods for fabricating CNT-reinforced MMCs, several emerging techniques show promise in addressing challenges such as uniform CNT dispersion, interfacial bonding, and the creation of composites with unique or complex properties. Some of the key emerging techniques that are gaining attention in the field are Laser Ablation, Spark Plasma Sintering (SPS), and electrostatic assembly.

SPS [197] is an advanced processing technique that provides better control over the formation of the composite. SPS uses pulsed electrical current to sinter the composite materials at a low temperature, reducing the risk of CNT degradation while ensuring strong bonding between the CNTs and the metal matrix [198].

Electrostatic assembly is an emerging technique that involves aligning CNTs within the metal matrix using electric fields [199]. This method is particularly useful for creating composites with well-organized CNTs, which can significantly enhance the mechanical properties of the composite. The alignment of CNTs ensures that they are oriented in the direction of stress application, improving the overall strength and toughness of the composite [200].

Additive manufacturing, commonly referred to as 3D printing, is rapidly emerging as a powerful tool for the production of CNT-reinforced MMCs with complex geometries and tailored properties [201]. This technique allows for better control over the distribution of CNTs, especially in composites that require high performance under specific conditions [202]. Moreover, additive manufacturing enables the production of parts with intricate designs, which is not possible with traditional fabrication techniques.

Electrochemical deposition is a technique that involves the electroplating of metal onto CNTs to form a uniform matrix [203]. This technique is particularly useful for the production of CNT-reinforced MMCs with high electrical conductivity and corrosion resistance, which are desirable in electronic and automotive applications [204]. The electrochemical deposition process provides precise control over the amount of metal deposited on the CNTs, ensuring uniformity and enhancing the bonding between the CNTs and the metal matrix.

The integration of CNTs into metal matrices has opened a pathway to develop advanced composites with superior mechanical, thermal, and electrical properties. For different metal matrices, various fabrication processes are employed to optimize performance. Below are the most relevant technologies categorized by the type of metal matrix.

To produce Aluminum Matrix Composites (Al-CNTs), Liu et al. [205] used powder metallurgy combined with a solution-assisted wet mixing technique to incorporate CNTs into an aluminum matrix. Ethanol was effective as a dispersing medium, enhancing CNT dispersion and minimizing agglomeration. The composites were synthesized with CNT volume fractions of 1.5%, 3%, and 7.5%. Tensile testing revealed a remarkable improvement, with yield strength increasing proportionally to the CNT content. Microstructural analysis using SEM and TEM confirmed uniform CNT dispersion and effective interfacial bonding, with minimal structural damage to the CNTs. The study utilized a shear-lag model to quantify the load transfer from the aluminum matrix to the CNTs, demonstrating an optimal balance between dispersion and interfacial strength.

Zare et al. [206] focused on the enhancement of mechanical properties in Al-CNTs via Equal Channel Angular Pressing (ECAP), a severe plastic deformation process. The composites were reinforced with 2 vol% CNTs and subjected to multiple ECAP passes at room temperature. The eight-pass ECAP process achieved significant grain refinement, reducing grain size to sub-micrometer levels. Yield strength and hardness increased by 30% using these composites compared to the use of pure aluminum. Fractographic analysis under SEM revealed that the failure mode shifted towards quasi-brittle behavior, attributed to the rigid reinforcement provided by CNTs. The study also highlighted that increasing the number of passes to more than eight offered no significant improvements and caused damage to the CNTs, ultimately reducing their reinforcing effect.

Rikhtegar et al. [207] compared two innovative approaches to integrate CNTs into aluminum matrices: semi-wet mixing and slurry-based dispersion. The slurry-based method, employing an aqueous medium and surfactants, provided superior dispersion and led to enhanced mechanical properties. For composites with 1.5 wt% CNTs, yield strength increased from 90 MPa (pure Al) to 152 MPa, and tensile strength rose from 136 MPa to 203 MPa. XRD and Raman spectroscopy confirmed the retention of CNT structural integrity post-processing, while microhardness tests showed uniform property distribution across the composite.

Esawi et al. [208] utilized high-energy ball milling to achieve a homogeneous dispersion of CNTs in the Al matrix, followed by spark plasma sintering at 500 °C. The method demonstrated that prolonged milling times resulted in partial CNT degradation, but controlled milling parameters (e.g., low energy and short durations) preserved CNT integrity. The composite with 2 wt% CNTs exhibited a 21% increase in tensile strength compared to pure Al, attributed to effective grain boundary strengthening and the Orowan mechanism. Electron backscatter diffraction (EBSD) analysis revealed minimal grain growth during sintering, ensuring high mechanical stability.

A dense AlSi-CNT composite was fabricated by Xie et al. [209] using an innovative combination of flake powder metallurgy and cold spraying. The flake morphology enhanced CNT dispersion within the AlSi matrix, while cold spraying ensured minimal CNT degradation. Tensile testing indicated a significant improvement in both yield strength and ductility, while microstructural analysis showed excellent CNT–matrix bonding. This method was particularly effective in producing complex geometries while retaining the mechanical benefits of CNT reinforcement.

Dhore et al. [196] implemented a blind mold sintering method to increase the strength of the CNT-reinforced aluminum matrix nanocomposites. The addition of CNTs and Sn powder to aluminum improved the composites’ hardness and wear properties. After the sintering process, the CNTs tended to retain their shape. The maximum hardness obtained was 73 BHN for 2 wt% Sn and 1 wt% CNTs. There was strong binding among Al-CNTs, which was observed through SEM images, and the dispersion was uniform.

Magnesium Matrix Composites (Mg-CNTs) were also investigated by Habibi et al. [210], who employed a microwave sintering process to fabricate Mg/Al–CNT composites, leveraging rapid heating to reduce grain size and improve bonding between the CNTs and Mg matrix. Al was introduced as a secondary reinforcement to enhance CNT dispersion and strengthen the matrix. The composite exhibited a 40% increase in tensile strength compared to pure Mg, attributed to a synergistic effect between Al and CNTs. TEM analysis revealed nanoscale intermetallic phases that contributed to the high mechanical performance.

To enhance CNT dispersion, Liang et al. [211] applied ultrasonic energy during the extrusion of AZ91D Mg alloy composites reinforced with CNTs. The ultrasonic vibrations disrupted CNT agglomerates and facilitated uniform distribution. Mechanical tests showed a 13.5% improvement in yield strength and a 25.5% increase in tensile strength. SEM images of fractured surfaces revealed a ductile failure mode, with CNTs bridging cracks and improving toughness.

Park et al. [193] used Si-coated CNTs to improve wettability and interfacial bonding in Mg composites. The squeeze infiltration method applied high pressure to infiltrate the Mg matrix into a preform of aligned CNTs. Tensile strength improved by 35%, and high-resolution microscopy revealed that the Si coating acted as a diffusion barrier, preventing CNT degradation during processing.

For the production of Copper Matrix Composites (Cu-CNT), Zheng et al. [203] utilized electrochemical deposition to fabricate Cu-CNTs with a uniform CNT distribution. By optimizing the deposition parameters, the authors achieved a 20% increase in microhardness with only 0.42 wt% CNTs. SEM and TEM analysis confirmed uniform CNT coating within the Cu matrix. The enhanced properties were attributed to the formation of a robust interfacial layer facilitated by electrostatic forces.

Xiong et al. [212] used a multi-step process involving spray pyrolysis and high-energy ball milling to reinforce Cu–Ti alloys with CNTs. Spray pyrolysis improved CNT wettability by depositing Ti on their surface, while ball milling ensured homogeneity. The resultant composite exhibited a 39% increase in tensile strength and a 62% improvement in ductility compared to pure Cu–Ti alloys. XPS analysis confirmed the chemical stability of CNTs after processing.

Chen et al. [213] focused on improving interfacial bonding by coating CNTs with Ni before incorporating them into the Cu matrix. SPS was employed at 600 °C to consolidate the composite. The resulting material exhibited a strong interfacial adhesion between the Ni layer and the Cu matrix. The yield strength of the composite with 2 vol % Ni-coated CNTs was 554 MPa, which was significantly higher than that of pure copper. The approximate interface shear strength in Ni-CNTs/Cu composites is 179 MPa, which is higher than the shear strength of the matrix.

## 6. Conclusions

The utilization of advanced fillers for the production of multifunctional composites is of great interest for the development of innovative solutions for aerospace. The carbonaceous fillers have been shown to be among the most outstanding materials for toughening polymeric and metal composites. CNTs stand as state-of-the-art carbon fillers that are able to improve both surface and bulk mechanical and electrical properties. The large-scale production of CNTs with standardized procedures has led to their adoption within the aerospace industry even if their price remains high. The improvements achieved by incorporating CNTs into composites are hardly attainable by using alternative species, thus highlighting their use in performance-oriented applications.

## Figures and Tables

**Table 1:** Mechanical, electrical, and thermal properties of SWCNTs and MWCNTs.

	Young Modulus [GPa]	Tensile Strength [GPa]	Resistivity [Ωm]	Thermal Conductivity [W/mK]
SWCNT	900–1700 [23]	75 [24]	10^−6^ [25]	1750–5800 [26]
MWCNT	690–1800 [27]	150 [24]	10^−5^ [28]	3000 [29]

**Table 2:** Summary of the main CVD production processes for CNTs with short descriptions and associated references.

Technique	Product	Advantage/Main Result	Ref.
Remote plasma CVD	SWCNTs	Selective production of SWCNTs	[39]
Aerosol CVD		Reduced sheet resistance	[40]
Spark-discharge aerosol CVD		Catalyst over-coagulation	[41]
Spark-discharge generator for scalable aerosol CVD		Independence between growth parameters and the diameter distribution	[42]
Continuous CVD process		Enhanced catalyst efficiency	[43]
CVD using monometallic and bimetallic catalysts		Bimetallic catalyst influence in chirality	[44,45]
CVD using sulfur		Increased yield	[46]
Hot filament reactor		Relation between density and diameter of grown SWCNTs	
CVD using different metallic supports		Strong relationship between the metallic support and both the type and quality of CNTs	[47,48]
Fischer–Tropsch-derived gas as carbon precursor		High-quality and well-aligned SWCNT carpet	[49]
Acrylonitrile base process		SWCNTs with uniform diameters and (n, m) chiral indices	[50]
Water-assisted CVD	MWCNTs	High yield	[51]
Acid-oxidized injection CVD		CNT diameter constant during growth	[52]
Microwave plasma CVD		Fast catalyst activation with energy savings	[53]
Thermal CVD on a nickel catalyst, deposited as substrates		Possibility to shift from producing CNTs to carbon fibers	[54]
Hot filament CVD		Long fibrils made by both MWCNTs and carbon fibers	[55]
Multimetallic catalyst CVD		Low-temperature CVD (600 °C)	[56,57,58]
Triode-type plasma-enhanced CVD		Length of up to 3 μm/temperature 550 °C	[59]
Iron and titanium as catalysts		Thin layer	[60]
Low-pressure CVD		Vertically aligned CNTs	[61]
